# Circadian rhythms in the blood–brain barrier: impact on neurological disorders and stress responses

**DOI:** 10.1186/s13041-023-00997-0

**Published:** 2023-01-12

**Authors:** Nicolette Schurhoff, Michal Toborek

**Affiliations:** 1 Department of Biochemistry and Molecular Biology, University of Miami Miller School of Medicine, Suite 528, 1011 NW 15th Street, Miami, FL 33155 USA; 2 Institute of Physiotherapy and Health Sciences, The Jerzy Kukuczka Academy of Physical Education, 40-065 Katowice, Poland

**Keywords:** Circadian rhythm, Blood–brain barrier, Brain, Neurological disorders, Stress responses

## Abstract

Circadian disruption has become more prevalent in society due to the increase in shift work, sleep disruption, blue light exposure, and travel via different time zones. The circadian rhythm is a timed transcription-translation feedback loop with positive regulators, BMAL1 and CLOCK, that interact with negative regulators, CRY and PER, to regulate both the central and peripheral clocks. This review highlights the functions of the circadian rhythm, specifically in the blood–brain barrier (BBB), during both healthy and pathological states. The BBB is a highly selective dynamic interface composed of CNS endothelial cells, astrocytes, pericytes, neurons, and microglia that form the neurovascular unit (NVU). Circadian rhythms modulate BBB integrity through regulating oscillations of tight junction proteins, assisting in functions of the NVU, and modulating transporter functions. Circadian disruptions within the BBB have been observed in stress responses and several neurological disorders, including brain metastasis, epilepsy, Alzheimer’s disease, and Parkinson’s disease. Further understanding of these interactions may facilitate the development of improved treatment options and preventative measures.

## Introduction to the circadian rhythm

The circadian rhythm is a “timekeeper” with rhythms of 24 h. The central clock is located in the suprachiasmatic nucleus (SCN) of the hypothalamus and its outputs regulate the peripheral clocks found throughout the body [1]. The central clock maintains an autonomous 24-hour rhythm, however, it is also influenced by external stimuli, termed “zeitgebers” (German for “time givers”); such as light that is intercepted and translated to the SCN by the retinohypothalamic tract composed of photosensitive retinal ganglion cells [2]. The SCN consists of a pair of nuclei that each holds approximately 10,000 neurons and 3,500 astrocytes that are situated above the optic chiasm [3]. The SCN can further be divided into two areas of function: the “core” ventrolateral area and the “shell” dorsomedial area. The SCN synchronizes peripheral clocks by executing neural control via the sympathetic and parasympathetic pathways as well as through humoral control [4, 5], such as the release of neuropeptides [6] [7–11] and the hypothalamic-pituitary-adrenal axis that regulates secretion of melatonin from the pineal gland and glucocorticoids and catecholamines from the adrenal cortex [12, 13].

The neurons that compose the SCN are mostly GABAergic, however, those in the “core” express vasoactive intestinal polypeptide (VIP), calretinin, gastrin related peptide, and neurotensin while those in the “shell” express arginine vasopressin (AVP), angiotensin II, prokineticin-2, and met-enkephalin [14]. The SCN neurons are unique within the mammalian brain as they form coupled, intercellular networks that carry out autonomous circadian oscillations of neuronal activity and gene expression [7, 15]. These intracellular communication networks are vital for synchronizing SCN activity between the “core” and “shell” [16]. VIP produced by “core” neurons has been suggested to influence other neuropeptides like AVP and gastrin-releasing peptide (GRP) by acting as a coupling signal [11, 17, 18]. VIP knockout was shown to desynchronize SCN activity and experimental addition of VIP induced phase shifts in the circadian rhythm cycle [17]. The SCN regulates circadian oscillations of molecular timing as demonstrated by oscillations in SCN electrical activity with high firing rates in light periods regardless of diurnal or nocturnal behavior [19]. Interestingly, a recent study showed that a specific population of mouse VIP positive SCN neurons are active during dark periods, opposite to most SCN neurons, and that this activity was vital for regulating nighttime sleep for mice between periods of activity. It was proposed that these neurons may increase ability to fall asleep due to sleep pressures by inhibiting activity and promoting quiescence. Alternatively, VIP positive neurons in the SCN may signal directly to promote sleep and fatigue. These findings suggest a wider role for SCN neurons than simply maintaining the 24-hour rhythms such as regulating critical minute features of the sleep-wake cycle.

**Fig. 1:**
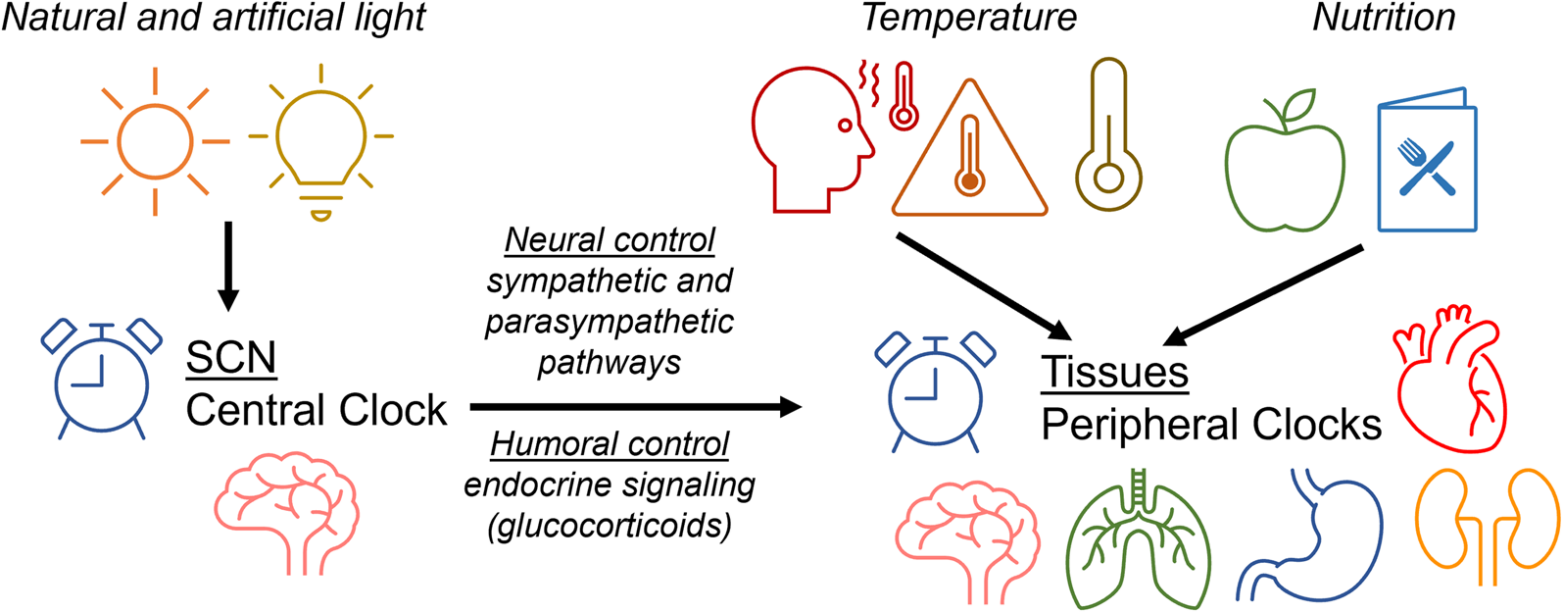
General interactions between the central clock and peripheral clocks. The central clock, located in the SCN, creates an autonomous circadian rhythm; however, signals from the environment termed “zeitgebers” (German for “time giver”) also influence these rhythms. In the SCN, the most common of these zeitgebers is light from both artificial and natural sources. Peripheral clocks are synchronized vertically by the SCN through neural and humoral pathways, and horizontally by zeitgebers such as temperature and nutrition

The peripheral clocks function to maintain rhythmicity in body temperature, metabolism, and hormone secretion (Fig. 1) [20, 21]. The circadian rhythm functions via a transcription-translation negative feedback loop, which contains positive regulators, such as transcription factors brain muscle aryl hydrocarbon receptor nuclear translocator-like 1 (BMAL1) and circadian locomotor output cycle kaput (CLOCK), as well as negative regulators that include cryptochrome-1/2 (CRY1,2) and period-1/2/3 (PER1,2,3) [22]. Peripheral clocks maintain 24-hour rhythmicity and are influenced by both the central clock and zeitgebers such as nutrition and temperature [16, 23, 24]. This allows the circadian rhythm to adjust to the surrounding environment and maintain rhythmicity. Analysis of 12 tissues’ protein coding genes found that 43% of them show circadian rhythms in transcription, mainly in an organ-specific manner. Furthermore, more than 1000 known and novel noncoding RNAs were demonstrated to express circadian oscillations as well [25]. When analysis was extended to 64 tissue and brain regions, more than 80% of protein coding genes were found to show circadian rhythms in transcription [26]. Mutations in circadian genes have been shown to correlate with various diseases such as an increased risk of type 2 diabetes, circadian sleeping disorders, and cancer [27]. These findings further emphasize the circadian rhythm’s importance and control over the transcriptome. The rise of circadian rhythm disruption within the population due to shift work, sleep disruption, travel via different time zones, and exposure to blue light devices has led to an increase in subsequent chronic diseases such as neurological disorders, cancer, metabolic disorders, mood disorders, alterations in intestinal integrity and gut microbial composition [28–31].

Interesting interactions of circadian rhythms with the gut microbiome in the context of maintaining tissue barrier integrity have recently been described. Upon circadian disruption, an increase in pro-inflammatory intestinal bacteria, concurrent with increased gut leakiness, were found in gut microbiota populations [31, 32]. Concurrently, gut microbiota was also shown to influence circadian rhythms through undergoing diurnal compositional and functional oscillations [33, 34]. Further suggestion for the circadian rhythm and gut integrity relationship are the abilities of circadian rhythms to control local and systemic metabolic process and inflammation responses [35–39]. Epidemiological studies have reported that people with frequent circadian disruptions, such as shift workers, have a higher incidence of sleep disturbances that lead to worsening of current health issues and potential future development of chronic disease [40–42]. Importantly, alterations of the gut microbiome have a profound impact on the BBB integrity via the brain-gut-microbiome axis [43, 44].

While the circadian rhythm has whole body effects and regulates several vital metabolic functions, this review focuses on the regulation of circadian rhythm in the cells of the blood–brain barrier (BBB) and its input on functional alterations of the BBB. In addition, we discuss circadian disruptions in various stress responses and neuropathological diseases that are associated with alterations of the BBB integrity.

## The molecular clock

**Fig. 2:**
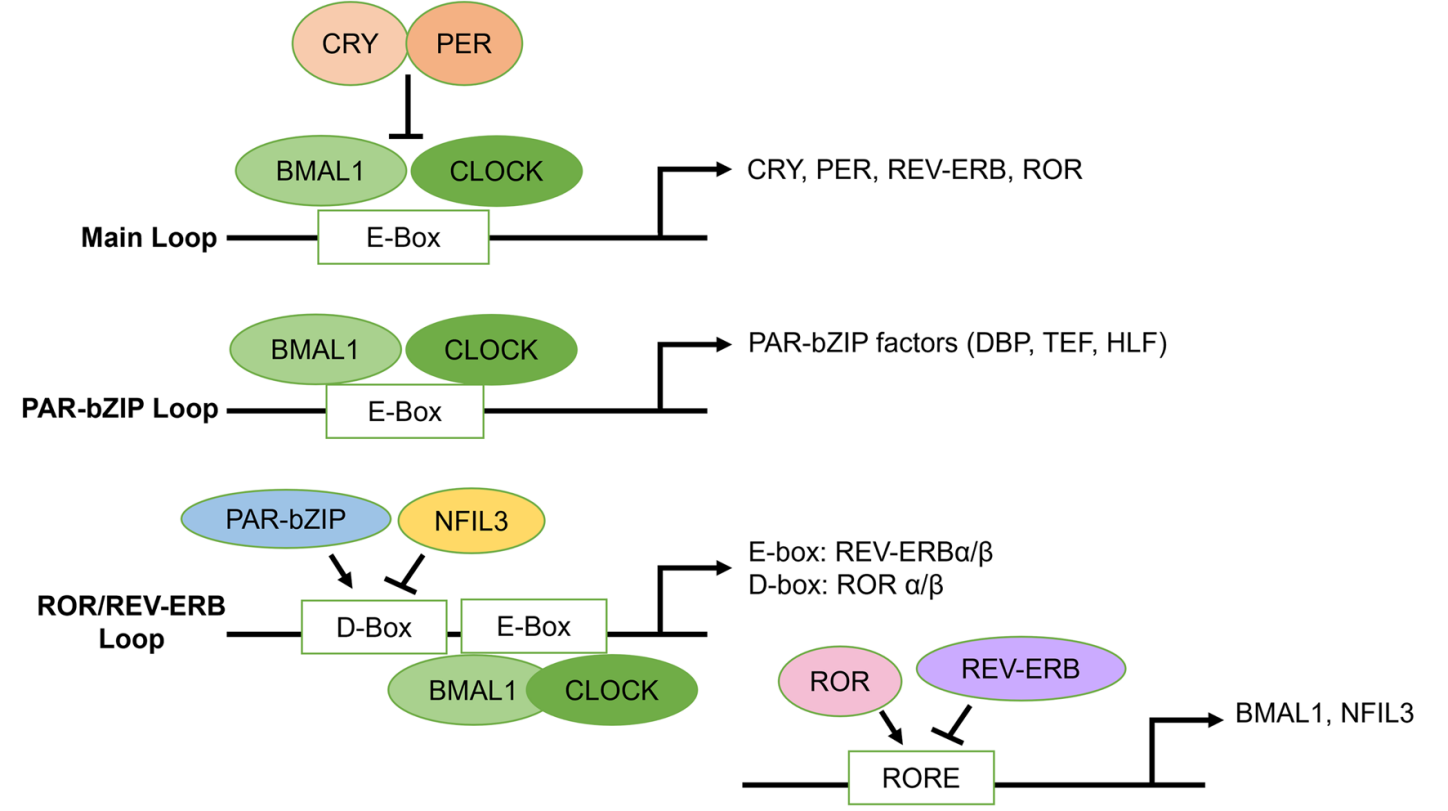
The molecular clock. The molecular clock is composed of the main loop, the ROR/REV-ERB loop, and the PAR-bZIP loop. In the main loop, circadian clock genes BMAL1 and CLOCK heterodimerize and bind to E-box regions to induce transcription of other circadian clock genes such as CRY and PER which upon accumulation, heterodimerize to inhibit their own transcription. CRY and PER are later targeted for proteasomal degradation. In the PAR-bZIP loop, BMAL1 and CLOCK bind to E-box regions to modulate the transcription of PAR-bZIP factors DBP, TEF, and HLF. In the ROR/REV-ERB loop, BMAL1 and CLOCK bind to E-box regions to promote transcription of REV-ERBα/β, which bind to RORE to inhibit BMAL1 and NFIL3 transcription. In addition, PAR-bZIP factors bind to D-Box regions in the ROR/REV-ERB loop to promote transcription of ROR α/β, which then binds to RORE to stimulate transcription of BMAL1 and NFIL3

The mammalian molecular clock is at its core a transcription-translation negative feedback loop (TTFL) that regulates circadian oscillations. At the start of the 24-hour cycle, positive regulators, such as BMAL1 and CLOCK, heterodimerize and bind to cis-acting enhancer elements to activate the expression of negative regulators, namely, transcription factors CRY and PER (Fig. 2) [22, 45]. As the cycle progresses, CRY and PER accumulate and heterodimerize to inhibit the BMAL1/CLOCK heterodimers from activating transcription [46]. The cycle is terminated when CRY and PER *de novo* transcription expression levels decrease below a threshold level, and the CRY/PERIOD complex is targeted for proteasomal degradation by E3-ubiquitin ligases, which release the BMAL1/CLOCK heterodimer complexes and allow the cycle to recommence [47–49]. Modes of translational regulation of the circadian TTFL include the mammalian/mechanistic target of rapamycin (mTOR) signaling pathway which remains in tune with the circadian clock. Indeed, mTOR actively signals in the photic entrainment pathway in the SCN, regulating autonomous clock in circadian oscillators, and influencing cross-communication between SCN neurons and other coupled circadian oscillators [50].

The expression of circadian clock genes is rhythmic in the SCN and peripheral clocks with peak times of CRY/PER mRNA levels in the circadian day, which rests in antiphase to BMAL1/CLOCK mRNA levels that peak in the circadian night. This rhythmic expression allows the protein products to reactivate the TTFL the next day [51], and controls circadian activity of the transcriptome [52–54]. The most important component of the main TTFL is BMAL1, as loss of BMAL1 leads to circadian arrhythmia, whereas singular losses in the other components can be substituted with other factors. For example, the loss of CLOCK functions can be substituted with its paralog neuronal PAS domain protein 2 (Npas2), while PER1 may compensate for PER2 knockout, with minor changes to overall rhythms [55–57].

Other components of TTFL are also involved in the mammalian molecular clock by targeting promoters and enhancers of circadian clock genes. The retinoic acid-related orphan receptor (RORs) and the REV-ERB loop are both regulated by the CLOCK/BMAL1; however, they also can modulate CLOCK/BMAL1 expression via a feedback mechanism [58–60]. Indeed, the ROR/REV-ERB loop stabilizes the molecular clock by interacting with BMAL1 and CLOCK, which constitute ROR/REV-ERB-response elements (RREs). ROR activates transcription, whereas REV-ERB inhibits transcriptome activity. PER2 synchronizes this loop by interacting with REV-ERBα that affects both positive and negative elements [61]. The proline and acidic amino acid-rich basic leucine zipper protein (PAR-bZIP) transcription factors D-box binding protein (DBP), thyrotroph embryonic factor (TEF), and hepatic leukemia factor (HLF) also form a loop with the main TTFL and are expressed in cyclic rhythms in the SCN. DBP specifically is activated by BMAL1/CLOCK and repressed by CRY/PER [62]. The repressor nuclear factor, interleukin 3 regulated (NFIL3), driven by RORE/REV-ERB loop, interacts with PAR-bZIP transcription factor loop by repressing DBP which in turn regulates ROR nuclear receptors [63]. These three TTFLs coordinate their activities to regulate the circadian rhythms both in the SCN and throughout the body by targeting specific genes *cis*-elements such as E-box, RORE, and D-box located in the promoters and enhancers of circadian-regulated genes [64] (Fig. 2).

## Circadian rhythms in the BBB

**Fig. 3:**
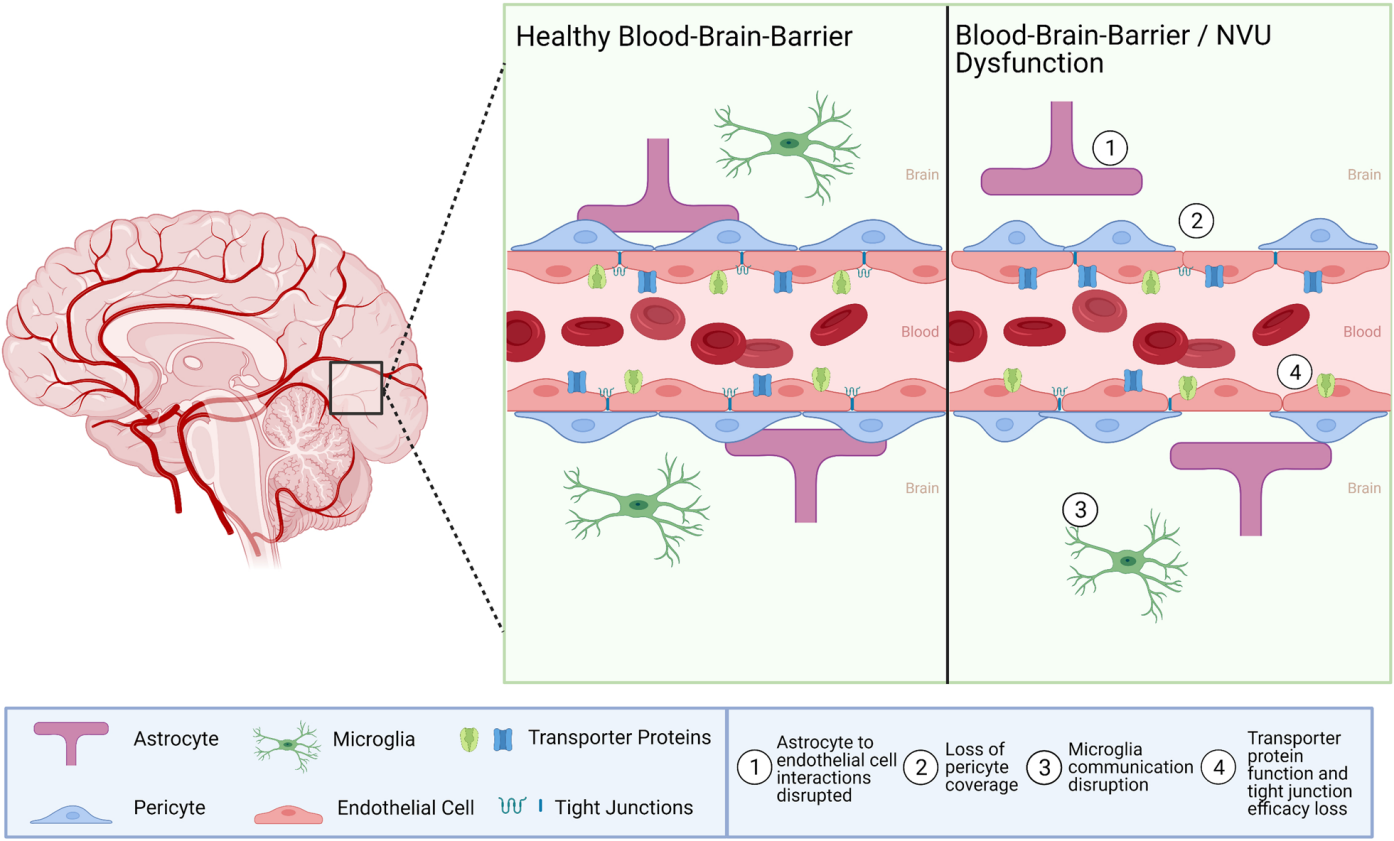
Circadian regulation of the BBB. The circadian rhythm regulates various aspects of BBB integrity such as tight junction efficacy, transporter activity, and NVU function. Under healthy conditions these functions work in harmony to maintain homeostasis and BBB integrity. However, when the circadian rhythm is disturbed signaling pathways become disrupted and loss of function or dysregulation ensues. Examples of such shown in the figure include (1) astrocyte to endothelial cell interactions disruption, (2) loss of pericyte coverage, (3) microglia communication disruption, (4) transporter protein function and tight junction efficacy loss. Created with BioRender.com

The BBB is a highly selective dynamic interface between the brain parenchyma and vascular system that separates the brain from the rest of the body (Fig. 3). The neurovascular unit (NVU) of the BBB is formed by the structural and molecular intercellular interactions between astrocytes, endothelial cells, and pericytes that interact with neurons and microglia. The NVU is critical in maintaining proper BBB barrier function through signaling pathways, including the Wnt/β-catenin pathway that regulates angiogenesis and barriergenesis [65]. The selectivity of the BBB is determined in part by endothelial tight junction proteins that form a physical barrier between the neighboring cells, while membrane transporters and other mechanisms of vesicular transport form a transport barrier [66]. Passage of molecules across the BBB may occur through several different mechanisms, including endocytosis, transfer through the plasma membranes controlled by ATP-binding cassette (ABC) transporters, or through paracellular diffusion, especially in the case of dysregulation of tight junction proteins [67]. The BBB protects neurotransmission in the CNS by restricting entry of peripheral inflammatory mediators, including cytokines and antibodies [68]. These mechanisms are, at least partially, regulated by the ABC efflux transporters, which actively pump out a large variety of blood-borne substances, like endogenous metabolites, proteins, or xenobiotics [69]. Several molecules, which CNS levels undergo circadian rhythmic oscillations, such as tumor necrosis factor alpha (TNFα), leptin, β-amyloid, delta-sleep inducing peptide (DSIP), and prostaglandin D2 (PGD2) are also likely timed to the rhythmic transport changes of the BBB [70–74]. In addition, norepinephrine shows circadian oscillations by accumulation in brain parenchyma during wakefulness and removal during rest [67].

### Endothelial cells

Endothelial cells and their tight junction proteins (e.g., occludin and claudin-5) are the main structural elements of BBB that determine its barrier function. Interestingly, tight junction proteins were found to be subjected to regulation by circadian rhythms both at the mRNA and protein levels. Occludin mRNA exhibits time-dependent oscillations in wild type mice, which are lost in circadian disrupted mice (Clock^Δ19/Δ19^) and result in loss of tissue barrier functions [75, 76]. Claudin-5 also appears to be under control of the central clock as demonstrated by the findings from RNAi-mediated knockdown of BMAL1 influence on inner blood-retina barrier integrity [77]. β-catenin, which shows regulatory effects on barrier integrity, was found to regulate both occludin and ZO-1 expression [78]. In support of these reports, circadian regulation of tight junction protein expression was also observed in intestinal cells [79].

ABC efflux transporters, which are highly expressed by BBB endothelial cells, can be regulated by both autonomous endothelial cell circadian clocks and neuronal activity. For example, expression and function of ABC efflux transporters was shown to be inversely correlated to neuronal activity in a diurnal rhythm fashion with higher rates of active transport during active periods and lower rates during rest periods. This process resulted in maintenance of neurochemical balance through lower rates of expression and function of ABC efflux transporters in endothelial cells during active periods to allow for transmission of information, and higher rates during the rest periods to allow for waste clearance [80]. Another mechanism of circadian regulation of the BBB transporter functions may be related to magnesium transporter TRPM7. Specifically, it was demonstrated that BMAL1 can bind to the promoter of TRPM7, resulting in cycling of transcription and translation of this transporter. Magnesium is a required cofactor for efficient efflux; therefore, changes in TRPM7 expression can also influence circadian regulation of the BBB [81]. Further evidence for circadian regulation of endothelial cells and the BBB was demonstrated at the end of sleep wake cycles where active ATP transporters and passive carrier-mediated transporters functions increase to allow for higher rates of passage across the BBB [67].

In vitro experiments with brain endothelial cells and astrocytes found rhythmic circadian/diurnal expression of circadian clock genes and cytochrome P450 epoxygenases, Cyp4 × 1 and Cyp2c11, which convert arachidonic acid to epoxyeicosatrienoic acids that are involved in the increasing blood flow to active neurons during a hyperemic response [82]. These results suggest a circadian explanation for the timing of cerebrovascular events such as stroke which typically occur more frequently in the early circadian day between 6 am and 12 pm [82, 83].

Finally, inflammatory processes in endothelial cells of the BBB can also be regulated, at least in part, by circadian mechanisms. For example, it was demonstrated that TNFα undergoes circadian rhythmic oscillations in BBB endothelial cells and activates the interleukin-15 (IL15) system [84]. While TNFα is a strongly proinflammatory factor, IL15 showed protection against neuroinflammation and circadian disruption as IL15 knockout mice showed significantly higher rates of both than wild type mice [84].

### Astrocytes

Astrocytes are glial cells that form the NVU of the BBB and have a variety of roles such as nutrient homeostasis, neurotransmitter recycling, immune signaling, regulation of neuronal synaptogenesis, protection against CNS inflammation, and BBB maintenance [85]. Astrocytes also make up a significant fraction of the SCN and assist as synchronizers in neuron signaling of circadian circuits [6]. In the SCN, astrocytes form a connected network through connexin 43 gap junctions to allow for quick communication [86]. Inhibitors of glial activity cause dysregulation of SCN neuronal firing and circadian controlled diurnal rhythms [87]. Circadian clocks have also been found in mammalian astrocytes showing cyclic ATP release and rhythmic expression of clock genes [88–90].

There is limited research conducted in regard to the circadian rhythm relationship with astrocytes in the BBB. Thus, this represents a potential area of novel research for future projects. Nevertheless, there have been reports on the involvement of astrocytes located in the brain parenchyma on circadian regulations. The mechanism by which astrocytes maintain rhythmicity in neuron firing was shown in vitro in co-culture experiments with γ-aminobutyric acid (GABA) mediated γ-aminobutyric acid type A (GABAA) receptor signaling [91]. Within the SCN, GABA is the most common neurotransmitter as it serves as a primary synchronizing signal through phase communication [92, 93]. Astrocytes express GABA receptors and transporters which release calcium upon activation [94, 95]. The intracellular concentration of calcium in SCN astrocytes was found to display circadian oscillations anti-phase to SCN neurons [96]. Increased intracellular calcium concentrations serve as metabolic markers associated with the release of glutamate [97]. In addition, this oscillatory astrocyte calcium release can couple with extracellular glutamate release activates GABAergic neurons to inhibit SCN neuronal activity during the circadian night [96]. In order to support this notion, studies on SCN astrocytes with BMAL1 deletion found dysregulation of circadian cycles via lengthening of time periods through GABA signaling [91, 98], which could be reversed by pharmacological modulation of GABAA-receptor signaling [91]. While astrocytes normally function to reabsorb neurotransmitters such as GABA from the synaptic cleft, this reuptake was also impaired in astrocytes with BMAL1 deletion, and higher levels of GABA were measured in the SCN of BMAL1 knockdown mice [91].

### Pericytes

Pericytes are located in the basement membrane of the brain microvasculature and their coverage is inversely correlated to BBB integrity [99, 100]. Pericytes have several functions in the BBB such as regulating blood flow in the brain microvasculature, movement of inflammatory cells, clearance of toxic waste products, modulating specific BBB gene expression rhythms in endothelial cells, and inducing polarization of astrocyte endfeet that surround the brain microvasculature [101, 102]. This represents an opportunity for future research to investigate both normal functioning of circadian rhythms with pericytes and the effects of circadian disruption on these interactions. One study of note investigated the impact of BMAL1 deletion on pericytes. BMAL1 deficient mice showed age-dependent loss of pericyte coverage that led to BBB hyperpermeability. Mechanistically, BMAL1 knockout led to downregulation of platelet-derived growth factor receptor ß (PDGFRß) transcription [103]. PDGFRß is both essential for pericyte recruitment and acts as a receptor for PDGFR-BB, whose coupled signaling in pericytes is essential for maintaining BBB integrity [104]. In addition, PDGFRß serves as one of the cellular markers of pericytes [105].

### Microglia

Microglia are phagocytic cells in the CNS that rapidly respond to neuroinflammation and tissue damage through increased phagocytic activity and cytokine production [106]. Microglia ablation in mice was found to strongly disrupt diurnal rhythms of several physiological processes and cause dysregulation of circadian clock genes and proteins in the SCN and hippocampus [107]. In addition, the circadian rhythms tightly control the microglial inflammatory responses. When the immune system response is initiated, microglia produce higher rates of pro-inflammatory cytokines, especially during light periods [108, 109]. Microglia also have a circadian dependency of response to glucocorticoids in stress-induced neuroinflammation that explains their diurnal rhythm response to inflammatory stimuli [108, 110]. A recent study described the circadian BMAL1-REV-ERBα axis as a regulator for complement (C4b and C3) expression and microglial synaptic phagocytosis in the brain [111]. The circadian rhythm is also involved in influencing microglia immunological activity as CLOCK is a positive regulator of NF-κB-mediated transcription, an innate immune system response which produces an immediate inflammatory reaction [112].

## Circadian rhythms and BBB-associated neurological disorders

**Fig. 4:**
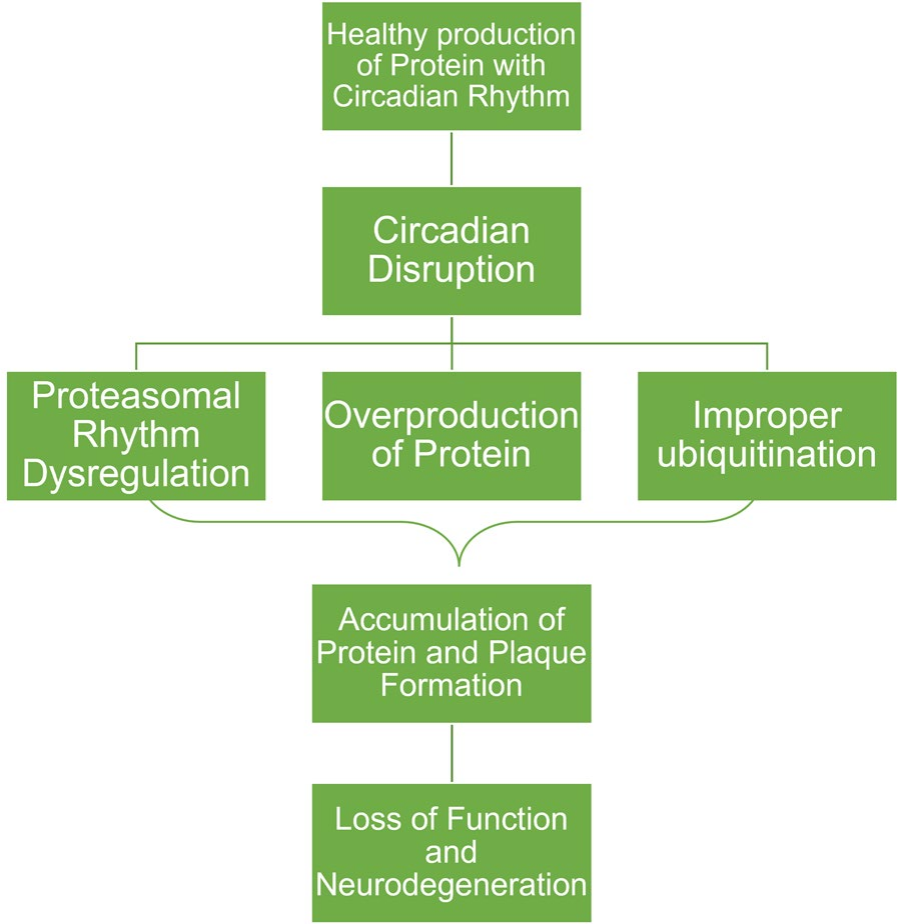
Circadian interactions in regulation of proteasomal activity. Proteasomal activity follows a circadian rhythm. Circadian disruption can result in rhythm of activity disruption, overproduction of proteins, and improper ubiquitination of inappropriate structures. Many neurodegenerative diseases involve an accumulation of these protein products that form meta-stable plaques, the formation of such leads to a loss of neuroprotection

Given the importance of the BBB in maintenance of the CNS homeostasis, and the interactions between circadian rhythms and the functional NVU, circadian disruptions have been implicated in the pathology of numerous chronic brain diseases. The impact of alterations of circadian rhythms on brain metastasis, epilepsy, Alzheimer’s disease, Parkinson’s disease, and stress disorders such as post-traumatic stress disorder will be discussed in the present review due to the strong link of these diseases to BBB and pathology of NVU **(**Fig. 4**)** [113–117]. However, circadian disruption has also been shown to correlate with other chronic diseases such as sleep disorders, tumorigenesis, psychiatric disorders, and a variety of neurodegenerative diseases [118–121]. The circadian rhythms effects range from molecular, cellular, physiological and behavior processes. Degradation of the SCN, followed by subsequent circadian disruption in the molecular clock, is prevalent in patients with Alzheimer’s disease, while degradation of dopaminergic striatal neurons which also affects circadian cycles is prevalent in patents with Parkinson’s disease [122]. Loss of BBB integrity is present in many neurodegenerative diseases such as Alzheimer’s disease and Parkinson’s disease [123]. Furthermore, drug transportation across the BBB poses challenges in treating these diseases as well. Many neurodegenerative diseases result from the accumulation of proteins that form meta-stable fibrils and amyloid aggregates. Often these proteins undergo circadian rhythms in their expression levels. Proper ubiquitination of inappropriate structures is key to maintaining a healthy microenvironment and preventing against neurodegenerative development and progression. Proteasomal activity follows a circadian rhythm and circadian disruption can negatively impact proteasomal activity leading to less neuroprotection against protein plaque formation [119].

### Brain metastasis

Brain metastases are the most common form of brain cancer that affect approximately 30–40% of cancer patient course of disease [124]. Brain metastases are difficult to treat due to location, barrier properties of the BBB, immune and/or metabolic constraints [125]. Therefore, new therapeutics including those that can cross the BBB are vital. Drug meditated circadian regulation may provide a novel outlet that leads to better outcomes. Indeed, circadian regulation of the BBB emphasizes the potential of chronotherapy in brain metastasis treatment. A study investigating the timing of chemotherapy admission based on circadian phase for treatment of brain metastases of breast cancer (BMBC) found that injections administered during the circadian dark phase increased BMBC cell death and delayed neurological symptom onset [126]. It has been demonstrated that the BBB undergoes remodeling upon crosstalk from brain tumor cells to form a more conducive tumor niche called the blood-tumor barrier (BTB) [127]. The transition from the BBB to the BTB causes a loss of BBB integrity, a shift towards a high heterogenous structure, and variable permeability changes [113]. In general, tight junction proteins of the BBB or BTB are considered to have a tumor-suppressive role as their decreased expression can lead to increased metastasis [128]. On the other hand, overexpression of tight junction proteins may have impact on promoting progression in cancers [129] and contribute to invasion and adhesion as demonstrated in human melanoma [130].

Mechanistically, circadian regulation has been found to interact with the cell cycle through circadian control of regulatory factors of the cell cycle such as Wee1, cyclin D1, and c-Myc [131]. Furthermore, BMAL1 and CLOCK interact with cyclin B1 to control cell cycle G2/M checkpoint, a vital regulatory step dysregulated in cancer. BMAL1/CLOCK knockout resulted in circadian disruption, reduced expression of cyclin B1, and lengthening of the cell cycle from delayed G2/M transition [132]. Thus, circadian dysregulation shows cell cycle changes that may be indicative of cancer [118].

A highly aggressive form of brain cancer, glioblastoma stem cells (GSC), was found to be dependent on central clock factors BMAL1 and CLOCK for growth and downregulation of these factors led to induced cell-cycle arrest and apoptosis [133]. Interestingly, circadian reprogramming was present in GSC as the circadian clock outputs were found to be redirected towards glucose metabolism and lipid synthesis, factors that aid in cancer progression [134]. Negative regulators of the core factors, CRY and REV-ERBs, when stimulated by small-molecule agonists, were found to downregulate stem cell factors and reduce GSC growth [133]. GSC reliance on BMAL1/CLOCK complex was found to be in part stimulated by downstream transcriptional upregulation of olfactomedin like 3 (OLFML3), a novel chemokine that functions to promote immune-suppressive microglia infiltration into the tumor microenvironment [135].

Circadian cell-cycle progression can also fluctuate radiosensitivity to cancer therapy as was demonstrated in a study that found gamma knife radiosurgery treatment for brain metastasis from nonsmall cell lung cancer were found to have better treatment outcomes if treatment was given earlier in the circadian day rather than later [136]. Another study found that glioma tissue with high expression rates of PER1 and PER2, which are commonly downregulated in cancer cells, were more susceptible to x-irradiation which induced apoptosis in these cells suggesting that circadian alteration may lead to better outcomes in radiotherapy [137]. Deregulated PER2 via methylation of promoters led to disruption of circadian rhythm, enhanced glioblastoma cell survival, and promotion of carcinogenesis [138]. Overall, most literature on implications of circadian rhythms with brain cancer, much less on brain metastasis, has been on time-of-day treatment outcome studies and glioblastomas. This represents an opportunity for future research investigations into circadian rhythm interactions with various brain cancer types and/or the ability of metastatic cells to traverse the BBB whether through direct regulation by core factors or indirect modulation of related TTFLs.

### Epilepsy

In terms of global burden of neurological diseases, epilepsy ranks third as the leading contributor and affects 65 million people worldwide. Treatment options are available such as single or multiple dose medication, resective surgery, neuromodulation devices, or dietary therapies, however, one-third of epileptic individuals continue to have seizures despite treatment [139]. BBB integrity also plays a role in epilepsy as acute breakdown of the BBB triggered seizures in patients undergoing BBB disruption for improved drug delivery [140]. Furthermore, degradation of the BBB was found in postmortem hippocampi from people with epilepsy [141]. Sleep deprivation is also associated with BBB breakdown and an increase in seizure frequency [67]. Many individuals with epilepsy have reported disruption in their sleep-wake cycles which further worsens symptoms [142, 143]. Circadian timing and sleep have been showcased in the timing of seizures for epileptic individuals as there is a reduced likelihood of seizure incidents later in the circadian day and an increased likelihood the morning following sleep deprivation [144, 145]. Indeed, one study investigating mesial temporal lobe epilepsy and circadian rhythm found altered spontaneous locomotor activity of circadian core genes in early post-*status epilepticus* and epileptic phases suggesting a role for seizures as a circadian zeitgebers, such as light, which was linked to activation of the hippocampal-accumbens pathway [146]. The periodicity of seizures may be attributed to circadian clock core genes’ ability to disrupt inhibition of neuronal firing and the oscillations of excitatory signals [146–148]. Circadian rhythm intervention for treatment may be a viable option; therefore, chronotherapy, has been suggested for treatment use in epilepsy [149].

Mechanistically, rhythmic cycles and steady-state levels of circadian genes such as BMAL1, CLOCK, PER1, REV-ERBα, and RORα were found to be dysregulated in epilepsy. In addition, alterations of pyridoxal metabolism, mTOR pathway, and redox state that are also associated with epilepsy are also influenced by circadian rhythms [150]. Mutations in the proteins comprising the mTOR pathway’s two multiprotein complexes mTORC1 and mTORC2 cause epilepsy and are termed mTORopathies [150]. In the BBB, rapamycin mTOR inhibitors were found to have a protective effect by reducing BBB leakage and inflammation [151]. Furthermore, phosphorylation of BMAL1 by an mTOR kinase, which promotes PER2 protein synthesis, can suppress mTORC activity [150]. In mouse models, neuronal BMAL1 and CLOCK disruption led to a lower seizure threshold indicating a higher likelihood of seizures incidents [152, 153]. CLOCK knockout specifically resulted in reduced dendritic spine formation and paroxysmal depolarization shift, both molecular hallmarks of epilepsy, caused by altered electrical function of neuronal microcircuits composed of excitatory pyramidal cells [152]. PER1 has been suggested to serve as a biomarker of epileptic progression as it shows key changes during the acute phases of epileptic events [150]. In mouse hippocampus following an acute epileptogenic insult, PER1 was significantly upregulated and upon repeated insults showed reduced steady-state mRNA and protein levels [154, 155]. The PARbZIP loop was also found to be implicated in epilepsy, as triple knockout of its factors DBP, HLF, and TEF led to development of lethal audiogenic seizures in animal models [62].

### Alzheimer’s disease

Alzheimer’s disease (AD) is a progressive neurodegenerative disorder and is one of the major causative factors of dementia that affects about 50 million people worldwide [156]. Many therapeutics have targeted amyloid-beta (Aβ) plaques upon discovery that they could be cleared from the brain [157]; however, they failed to improve clinical outcomes. Therefore, more rigorous and additional treatment methodologies are needed to actively prevent loss and restore cognitive function. In the later stages of AD, the worsening of behavioral symptoms upon the day ending, termed ‘sundowning’, has been associated with AD induced phase shifts in the normal circadian functions of alertness [158, 159]. Alterations of the BBB were reported to contribute to AD pathology and/or hamper its therapy. For example, the BBB was postulated to regulate Aβ homeostasis as an interface contributing to Aβ accumulation in the brain [160]. Impaired BBB function, such as loss of tight junction integrity, leads to reduced Aβ clearance, increased circulating Aβ levels, and processing of Aβ precursor proteins [161]. Furthermore, promotion of Aβ generation has been linked to BBB dysfunction via stimulation of neuroinflammatory signals and oxidative stress that intensify β-secretase and γ-secretase activity [162]. It was demonstrated that the receptor for advanced glycation end products (RAGE) can mediate Aβ transport across the BBB and accumulation in the brain [163]. Similarly, RAGE was shown to be involved in accumulation of Aβ in brain endothelial cells, the main structural component of the BBB [163, 164].

Aβ has been known to demonstrate circadian rhythmicity for over a decade [165]. The PSEN2 gene, a regulator of Aβ levels, shows rhythmic oscillations in the SCN and is controlled by CLOCK/BMAL1 heterodimer both by transcriptional and post-translational mechanisms in peripheral tissues [52, 166, 167]. In addition, Aβ clearance across the BBB was shown to occur in a circadian oscillatory manner and increased during sleep [168]. This circadian oscillatory manner is related to circadian induced changes in cerebrospinal fluid movement in brain that promote waste clearances such as those seen in the glymphatic system [169]. Aβ accumulation was shown to disrupt the molecular clock and induce changes in molecular bioenergetics and metabolic circadian oscillations [170]. Specifically, Aβ induced post-translational degradation of BMAL1 which led to reduced binding to the PER2 promoter and subsequent disruptions of PER2 transcription and translation [171]. Oxidative stress from increased reactive oxidative species (ROS) and reactive nitrogen species (RNS) within the CNS has been suggested to promote AD development and progression [172]. Circadian disruption has been linked to increased oxidative stress in neurons leading to lipid peroxidation and protein and nucleic acid oxidation which is involved in the early pathogenesis of AD [173]. Loss of BMAL1 and CLOCK resulted in impaired expression of several redox defense genes and excessive production of ROS which led to chronic oxidative stress and neuronal oxidative damage [174, 175]. It was also demonstrated that BMAL1 deletion led to acceleration of Aβ plaque formation in central rhythms and promoted fibrillar plaque deposition in peripheral rhythms [72].

In addition to amyloidopathy, the pathogenesis of AD also involves an accumulation of hyperphosphorylated tau proteins that form intracellular neurofibrillary tangles [176]. Tau phosphorylation also follows a circadian rhythm driven by sleep and circadian induced changes in body temperature [177]. A study on a Tg4510 mouse model of pathological tau physiology, taupathy, found alterations in circadian rhythm at both the molecular and behavioral level. At the molecular level, taupathy was observed in parallel with disrupted PER2 rhythmicity in hypothalamus and PER2 and BMAL1 in the hippocampus [178]. The role of BMAL1 and circadian dysregulation in AD pathogenesis was confirmed in a recent study that demonstrated rhythmic DNA methylation associated with rhythmic BMAL1 transcription which was dysregulated during early AD progression [179].

### Parkinson’s disease

Parkinson’s disease (PD) is a progressive neurodegenerative disease that affects 2–3% of the population aged 65 years and older. It is characterized by neuronal loss in the substantia nigra leading to striatal dopamine deficiency and α-synuclein accumulation as deposits termed ‘Lewy bodies’ [180]. In addition, alterations of the BBB integrity have been associated with the progression of PD [116, 181, 182]. Circadian disruptions have been observed in both patients with PD and animal models along with PD symptoms exhibiting diurnal fluctuations [183]. In patients with PD, insomnia is one of the most common sleep disorders, reported by approximately 50% of patients [184, 185]. Patients with PD also showed reduced amplitude in rest/activity rhythm tied to circadian sleep wake cycles and reduction in both diurnal activity and nocturnal rest [186–188]. Circadian centered therapeutics have been effective in treating PD by improving both non-motor and motor symptoms, specifically bright light therapy which is typically used to reduce depressive symptoms in mood disorders such as seasonal affective disorder [189, 190].

Mechanistically, the molecular clock has been shown to impact various aspects of PD pathology, including dopamine production. In fact, dopamine synthesis is controlled by circadian clock genes as CLOCK regulates the rate-limiting enzyme, tyrosine hydroxylase, for dopamine synthesis [191]. CLOCK accomplishes this by binding to E-box elements located in the promoter regions of dopaminergic pathway related genes to regulate the transcription of tyrosine hydroxylase, dopamine activity transporter, and D1 receptor [192]. Dopaminergic activity can also be regulated by circadian genes as knockdown of CLOCK in the ventral tegmental area through RNA interference led to increased activity [193]. Alternatively, dopaminergic activity can also regulate circadian genes, specifically CLOCK, in a receptor-dependent manner [194]. This is achieved by upregulating transcriptional activity of the CLOCK/BMAL1 heterodimer via activating enhancer element cAMP responsive element-binding proteins [195]. PER2 rhythmic expression can be modulated by D2 receptor activation except for in the SCN [196]. Interestingly, D1 receptor agonists enhance expression of PER1, CLOCK, and BMAL1; however, D2 receptor agonists inhibit the expression of CLOCK and PER1 [194]. Overall, these findings suggest the circadian disruption in PD may result from dysregulation between the interactions of the dopaminergic and circadian pathways.

The involvement of circadian alterations in PD pathogenesis and disease progression has been confirmed in a study that found disruption of circadian genes in patients with PD. For example, BMAL1 mRNA expression in the peripheral leukocytes of these patients was significantly lower in the evening and BMAL1 levels correlated with motor severity and sleep quality [197]. In addition, BMAL1 single-nucleotide polymorphisms (SNPs) were associated with the risk for tremor dominant subtype while PER1 SNPs were associated with gait difficulties and postural instability dominant subtype [198].

### Stress responses and post-traumatic stress disorder

**Fig. 5:**
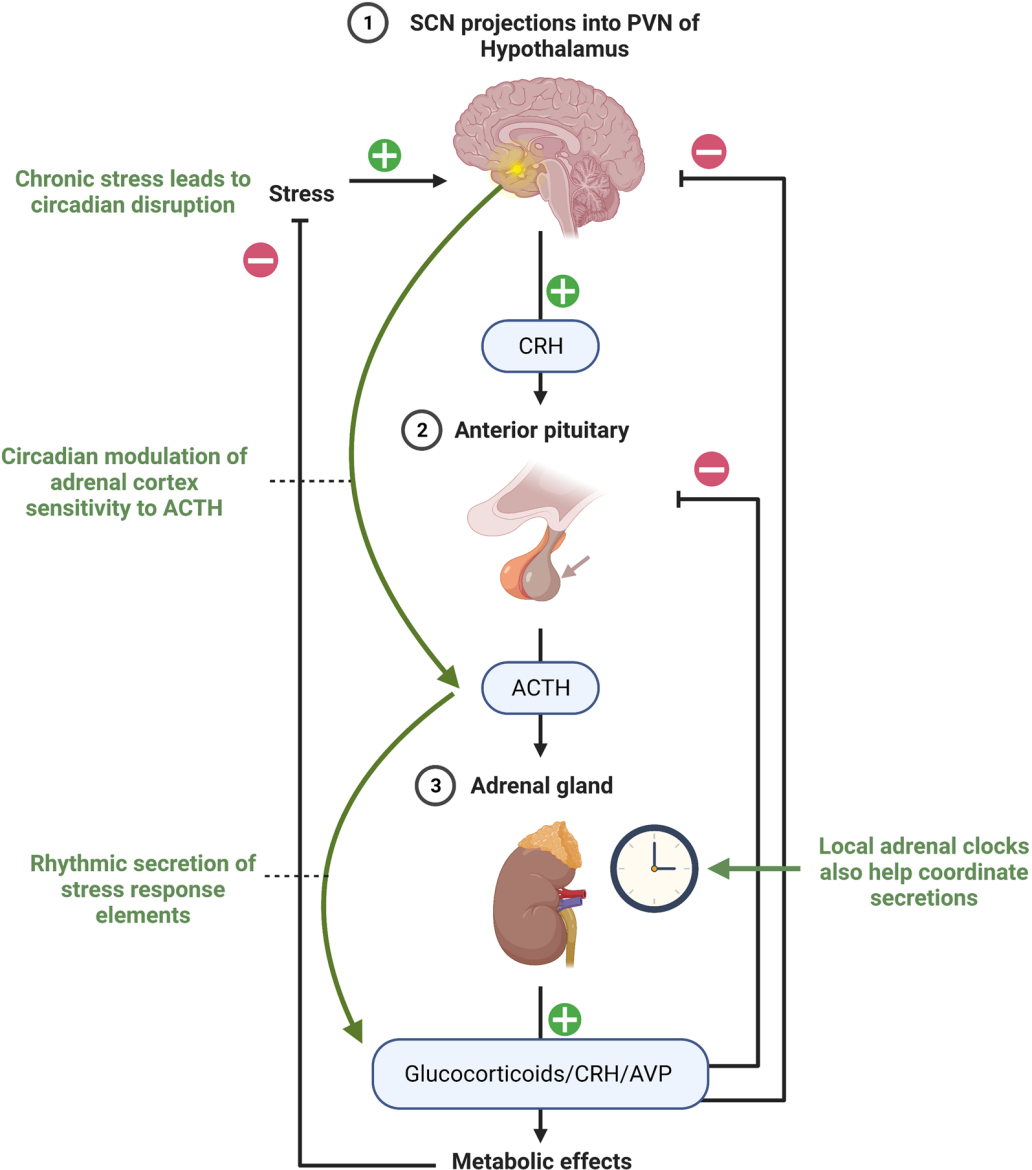
Circadian rhythm and stress response system interactions. The circadian central clock regulates the hypothalamic-pituitary-adrenal axis via projections from SCN that are sent into the paraventricular nucleus (PVN) in the hypothalamus. Stress prompts the release of corticotropin-releasing hormone (CRH) from the hypothalamus. CRH then stimulates the release of adrenocorticotropic hormone (ACTH) from the anterior pituitary gland. Stress response factors, such as glucocorticoids, CRH, and arginine vasopressin (AVP) (also known as antidiuretic hormone (ADH) are secreted rhythmically as signaled by the adrenal cortex whose sensitivity to ACTH is under circadian control. Local adrenal clocks also contribute to the rhythmic secretions and utilize BMAL1 to regulate steroidogenesis. Elevated levels of glucocorticoids due to stress can negatively impact the circadian central clock and dysregulate peripheral clocks. Created with BioRender.com

Stress responses, including post-traumatic stress disorder (PTSD), are known to be associated with disruption of BBB integrity [117, 199]. Moreover, the circadian rhythm and stress response system are interconnected through clock genes and endocrine signaling (Fig. 5) [200]. As presented in the Introduction section, the central clock regulates the hypothalamic-pituitary-adrenal axis via projections from SCN that are sent into the paraventricular nucleus (PVN) in the hypothalamus. This allows circadian regulation of the adrenal cortex sensitivity to adrenocorticotropic hormone which accounts for the rhythmicity seen in the secretions of stress response factors such as glucocorticoids, corticotropin-releasing hormone, and AVP [201, 202]. Specifically, the SCN sends information via photic transmission through the splanchnic nerve innervation to signal the adrenal glands to increase glucocorticoid release [203]. Local circadian clocks in the adrenal glands account for the rhythmicity in expression of about 10% of the adrenal genome [204]. In local adrenal clocks, circadian rhythm influences steroidogenesis through BMAL1 which regulates the transcription of steroidogenic acute regulatory protein, a rate-limiting gene encoding cholesterol transporters into the mitochondria [205]. These interactions are bidirectional as glucocorticoids can influence circadian rhythm through interactions with peripheral clocks that alter expression of PER [206]. Elevated glucocorticoids levels from chronic stress leads to reduced expression of PER in the SCN and impact functioning of the central clock and dysregulation of peripheral clocks [207].

Feedback between the circadian rhythm and stress response system has impact on increasing risk of onset for stress disorders such as PTSD, a psychiatric disorder that is caused by a previously experienced traumatic event and frequently initiates the stress response system [208]. As described earlier, circadian rhythm regulates various aspects of the stress response system; therefore, circadian disruption can lead to further dysregulation of stress responses. Indeed, interleukin 6, a pleiotropic pro-inflammatory cytokine elevated during psychological and physical stress, displays circadian oscillations across the BBB that differs in periodicity and function dependent on veteran stress exposure [209]. Moreover, elevated levels of glucocorticoids can promote neurotoxic stress on the hippocampus, a brain region implicated in PTSD [210, 211]. In animal studies, susceptibility to fear conditioning (an experimental model for PTSD) and corticosterone release were increased upon circadian disruption [212]. In addition, animal models of PTSD were found to have significant circadian disruption in the rhythmic secretions of serotonin, an important neurotransmitter in PTSD [213]. Within the brain, the amygdala has been shown to be implicated in PTSD manifestation and monitoring [213–215.

Genetic studies in humans have found several pathways that link circadian rhythm to stress vulnerability and PTSD. One of them appears to be FKBP5, a chaperone protein that guides activated glucocorticoids to the nucleus, which shows rhythmic expressions in most tissues, confirming circadian regulation of glucocorticoid signaling. FKBP5 has been implicated in several stress-related psychiatric disorders including PTSD [216, 217]. Genome wide association studies have found pituitary adenylate-cyclase-activating polypeptide (PACAP) and RORA, two core clock genes, to be implicated in PTSD as well. PACAP influences the central clock by phase resetting from incoming light zeitgebers [218]. PACAP is encoded by ADCYAP1 which is associated with increased amygdala and hippocampus response to fear [219]. RORA is involved in the REV-ERB loop that influences the molecular clock and has been identified as PTSD risk gene [220, 221]. Another gene loop involved in the molecular clock, a PARb-ZIP factor, TEF (variant rs5758324), showed strong associations with PTSD symptoms [222]. These findings further emphasize wide reaching implications involved in circadian disruption.

## Conclusion and future perspectives

Circadian rhythms guide various physiological processes such as the sleep-wake cycle, core body temperature, and metabolic pathways. These processes are regulated by both the central and peripheral molecular clocks that synchronize with one another through TTFLs and neuromodulators and are influenced by various zeitgebers. Within the brain, circadian rhythms interact in the central clock located in the SCN as well as with cells forming the NVU of the BBB that are vital for BBB integrity and maintaining brain homeostasis. When the circadian rhythm is disrupted either by environmental or disease means, the resulting alterations of downstream pathways of the molecular clock can further worsen or lead to development of chronic disorders. Patients with neurological disorders commonly suffer from circadian disruption and altered sleep-wake cycles which worsen conditions over time. On the other hand, chronotherapy, the timing of drug administration dependent on the phase of the circadian rhythm to achieve maximal benefit, has been suggested for treatment of various diseases involving circadian disruption [28, 223]. Indeed, the benefits of chronotherapy have already been demonstrated in Parkinson’s disease, epilepsy, cardiovascular disease, and hypertension [149, 224–226]. Research studies both in vivo and in vitro have elucidated a number of important functions of the circadian clock in various diseases, however, more research is needed to apply practically. Further research studies are warranted to delve deeper into the molecular interactions involved between circadian rhythms and neurological disorders to create better treatment plans and preventative measures.

## Data Availability

Available upon request.
